# Exogenous Application of Methyl Salicylate Induces Defence in Brassica against Peach Potato Aphid *Myzus persicae*

**DOI:** 10.3390/plants12091770

**Published:** 2023-04-26

**Authors:** Jamin Ali, Dongming Wei, Mohammad Mahamood, Fanrui Zhou, Patricia Jie Hung King, Wenwu Zhou, Imran Haider Shamsi

**Affiliations:** 1 School of Life Sciences, Keele University, Keele, Staffordshire ST5 5BG, UK; j.ali@keele.ac.uk; 2 Key Laboratory of Crop Germplasm Resource, Department of Agronomy, College of Agriculture and Biotechnology, Zhejiang University, Hangzhou 310058, China; 22016025@zju.edu.cn; 3 Department of Biology, Qassim University, Buraydah 51452, Saudi Arabia; m.mahamood@qu.edu.sa; 4 Department of Food Science and Nutrition, Zhejiang Key Laboratory for Agro-Food Processing, Zhejiang University, Hangzhou 310058, China; 12213077@zju.edu.cn; 5 Key Laboratory of State Forestry and Grassland Administration on Highly Efficient Utilization of Forestry Biomass Resources in Southwest China, College of Material and Chemical Engineering, Southwest Forestry University, Kunming 650224, China; 6 Institute of Ecosystem Science Borneo, University Putra Malaysia, Bintulu 97000, Malaysia; patricia@upm.edu.my; 7 Faculty of Agricultural and Forestry Sciences, University Putra Malaysia, Bintulu 97000, Malaysia; 8 Key Laboratory of Biology of Crop Pathogens and Insects of Zhejiang Province, Institute of Insect Sciences, Zhejiang University, Hangzhou 310058, China

**Keywords:** induced defence, methyl salicylate, tritrophic interaction, *Myzus persicae*, *Diaeretiella rapae*

## Abstract

Plants use a variety of secondary metabolites to defend themselves against herbivore insects. Methyl salicylate (MeSA) is a natural plant-derived compound that has been used as a plant defence elicitor and a herbivore repellent on several crop plants. The aim of this study was to investigate the effect of MeSA treatment of *Brassica rapa* subsp. *chinensis* (‘Hanakan’ pak choi) on its interactions with peach potato aphids, *Myzus persicae,* and their natural enemy, *Diaeretiella rapae*. For this, we selected two concentrations of MeSA (75 mg/L and 100 mg/L). Our results showed that aphid performance was significantly reduced on plants treated with MeSA (100 mg/L). In a cage bioassay, the MeSA (100 mg/L)-treated plants showed lower adult survival and larviposition. Similarly, the MeSA (100 mg/L)-treated plants had a significantly lower aphid settlement in a settlement bioassay. In contrast, the *M. persicae* aphids did not show any significant difference between the MeSA (75 mg/L)-treated and control plants. In a parasitoid foraging bioassay, the parasitoid *D. rapae* also did not show any significant difference in the time spent on MeSA-treated and control plants. A volatile analysis showed that the MeSA treatment induced a significant change in volatile emissions, as high numbers of volatile compounds were detected from the MeSA-treated plants. Our results showed that MeSA has potential to induce defence in *Brassica* against *M. persicae* and can be utilised in developing sustainable approaches for the management of peach potato aphids.

## 1. Introduction

Being in continuous co-evolution with insects, plants have evolved a broad armoury of effective and sophisticated defence mechanisms that make them more resistant to defend themselves against these invading insects accordingly before they cause significant damage [1]. Some of these chemical defences are constitutive but many are induced soon after the invasion of herbivores [2]. Interestingly, these inducible defences can be also achieved using bioactive chemical compounds, so-called elicitors or inducers of plant defence [3,4]. Such a strategy has pushed the idea that plant inducible defences can be improved upon or modified by turning on key genes responsible for the production of defence compounds [5,6].

Methyl salicylate (MeSA) is a natural compound generally released by plants under attack by herbivores, also known as a herbivore-induced plant volatile (HIPV) [7,8]. The effect of the MeSA compound has been tested in different forms, such as direct applications, trap cards, and lures [9,10,11]. Most of these studies proved that MeSA has a repellent effect on pests and pathogens; it reduces pest performance by altering their behaviour, oviposition, and colonisation [12,13]. Earlier studies have reported that MeSA has the potential to activate plant defence against herbivorous insect pests, such as cabbage moths (*Mamestra brassicae* L.) [14,15,16], bird cherry oat aphids (*Rhopalosiphum padi* L.) [17], and hope vine aphids (*Phorodon humuli)* [18]. MeSA has shown considerable potential in inducing plant defence against insect herbivores by inducing the synthesis of antibiotic and defensive compounds [16,19,20].

The current study was performed to test the effect of exogenous applications of MeSA on *Brassica rapa,* which is one of the important economical crops in the world. However, due to limited options for pest control, this crop is highly susceptible to herbivorous pests. At present, the effect of MeSA treatments of pak choi against *Myzus persicae* is unknown and no study has been performed to investigate the effect of MeSA treatments on *B. rapa* subsp. *chinensis* (Hanakan). Previous studies have reported that MeSA has been found to be repellent to herbivorous pests belonging to the family Aphididae (*R. padi* and *Phorodon humuli*) and Lepidoptera (*M. brassicae*). In this study, we hypothesised that MeSA treatments of pak choi will affect the performance of *M. persicae* in a similar way. Therefore, the present study was designed to test the hypothesis that MeSA treatments will reduce the performance and colonisation of *M. persicae* and increase parasitoid foraging time on treated plants. To test this hypothesis, performance and behavioural bioassays as well as headspace sampling were performed. This study is timely because sustainable approaches to protect brassica crops are required due to the banning of neonicotinoids and development of *M. persicae*’s resistance to most available insecticides.

## 2. Results

### 2.1. Aphid Performance Bioassay

**Figure 1:**
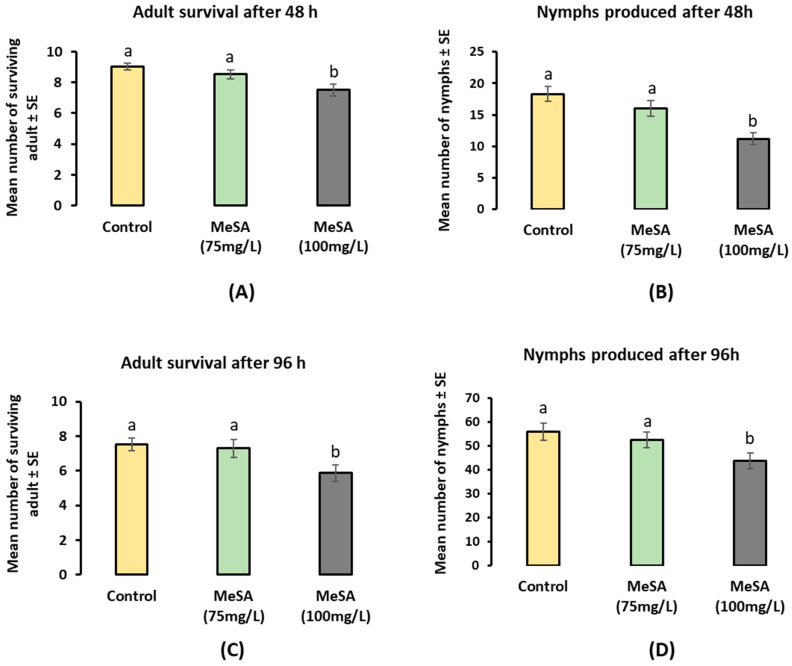
*Myzus persicae* (Mean ± SE): (**A**) adult survival after 48 h; (**B**) nymphs produced after 48 h; (**C**) adult survival after 96 h; and (**D**) nymphs produced after 96 h in clip cages on pak choi plants treated with different treatments (i.e., control, MeSA 75 mg/L and MeSA 100 mg/L; *n* = 15). Treatments labelled with different letters indicate statistically significant differences among plant species (F-test; *p* < 0.05) based on the Holm–Sidak method (one-way ANOVA).

In the clip cage bioassay, there was a significant reduction in the number of surviving aphids on the MeSA-treated plants compared to those on the control plants across both series 1 and series 2. After 48 h, the number of surviving *M. persicae* had decreased on the MeSA-treated plants (*F*_2,87_ = 6.764; *p* = 0.0018) (Figure 1A). In particular, an increase in mortality was recorded with an increase in concentration after 48 h: 9.7% (blank formulation), 14.7% on MeSA (75 mg/L), and 25% on MeSA (100 mg/L)-treated plants (Figure 1A). Similarly, after 96 h, lower numbers of surviving adults were recorded from the treated plants compared to the control (*F*_2,87_ = 6.764; *p* = 0.0248) (Figure 1C). In particular, an increase in mortality was recorded with the increase in concentration after 96 h, which showed a mortality of 24.7% (blank formulation, 27% on MeSA (75 mg/L) and 41% on MeSA (100 mg/L)-treated plants) (Figure 1C). However, the effectiveness of the MeSA on *M. persicae*’s performance was reduced after 96 h (*p* = 0.0248) compared to that after 48 h (*p* = 0.0018).

A similar pattern was observed for the larviposition; the plants treated with MeSA had less larviposition compared to that of the control plants. After 48 h, a significant reduction in larviposition was recorded between the blank formulation and MeSA-treated plants (*F*_2,87_ = 10.98; *p* = 0.000056) (Figure 1B). An overall larviposition reduction of 26% was recorded in the plants treated with MeSA, which varied according to the concentration (i.e., a 15% reduction on plants treated with 75 mg/L, while a 36% reduction on plants treated with 100 mg/L). Our observations recorded after 96 h also showed a significant reduction in larviposition across the control and treated plants; again, the intensity of the MeSA-induced effect on *M. persicae* was reduced after 96 h (*F*_2,87_ = 3.1536; *p* = 0.047619) (Figure 1D).

### 2.2. Aphid Settlement Bioassay

**Figure 2:**
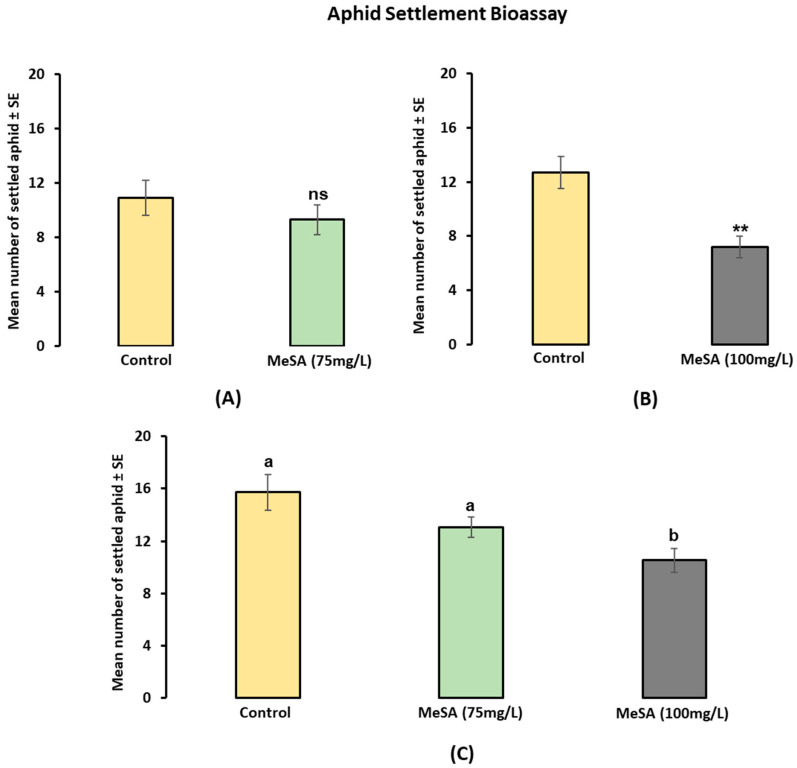
Settlement of *Myzus persicae* (Mean ± SE) after 24 h: (**A**) in series 1 (control vs. MeSA 75 mg/L; *n* = 10); (**B**) series 2 (control vs. MeSA 100 mg/L; *n* = 10); and (**C**) series 3 (control vs. MeSA 75 mg/L vs. MeSA 100 mg/L; *n* = 15). Treatments labelled ‘ns’ do not show a significant difference between control and MeSA concentrations while the asterisks indicate significance (**, *p* < 0.001 (*t*-test). Treatments labelled with different letters denote differing levels of statistical significance (F-test; *p* < 0.05) based on the Holm–Sidak method (one-way ANOVA).

In the settlement bioassays, there were three series of experiments; in series 1 (control vs. MeSA (75 mg/L)), no significant difference was observed in aphid settlement on the treated and control plants (*p* = 0.3480; paired *t*-test) (Figure 2A). In series 2 (control vs. MeSA (100 mg/L)), a significantly lower number of aphids had settled on the treated plants compared to the control plants (*p* = 0.0018; paired *t*-test) (Figure 2B). In series 3 (control vs. MeSA (75 mg/L) vs. MeSA (100 mg/L)), a statistically significant reduction in aphid settlement was observed on the MeSA-treated plants (*F*_2,42_ = 69,788; *p* = 0.006465) as shown by the one-way single-factor ANOVA (Figure 2C).

### 2.3. Parasitoid Fly-Off Bioassay

**Figure 3:**
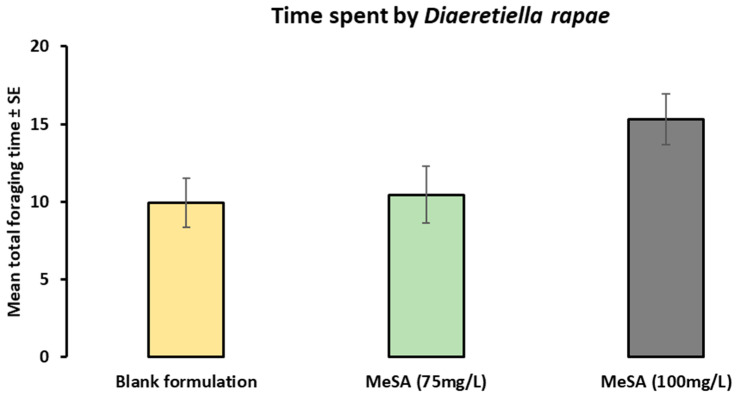
Mean total time (in minutes) spent foraging (Mean ± SE) by *Diaeretiella rapae* on the control and MeSA-treated plants (*n* = 15) (F-test; = 3.12814 *p* > 0.05), based on the Holm–Sidak method (one-way ANOVA). Parasitoid *D. rapae* did not show any significant differences in the time spent on MeSA-treated and untreated plants.

In the fly-off bioassay, the parasitoid wasps did not show statistically significant differences in the time spent on the blank formulation and MeSA-treated plants (*F*_2,42_ = 3.12814; *p* = 0.05415) as shown by a one-way single-factor ANOVA (Figure 3). In particular, the mean total time spent in minutes was 9.94 on the control plants, 10.44 on the (75 mg/L) MeSA plants, and 15.30 on the (100 mg/L) MeSA plants.

### 2.4. Volatile Analysis

**Figure 4:**
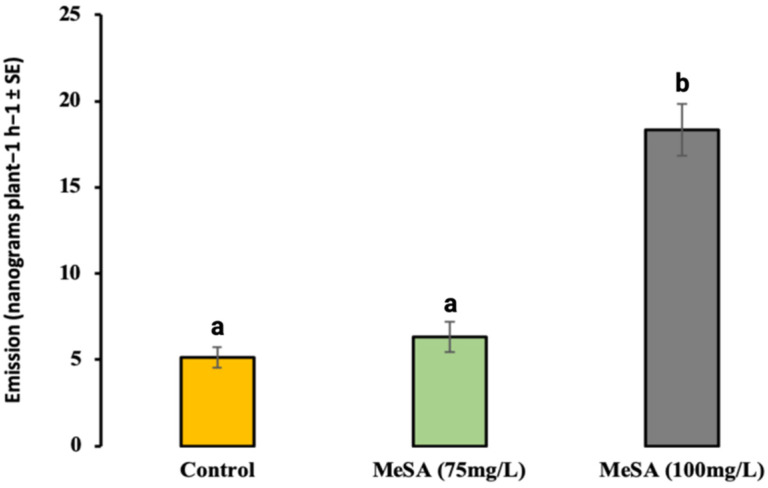
Total amount (mean nanograms per plant^−1^ h^−1^ ± SE) of identified VOCs emitted from *Brassica rapa* for treatments with MeSA and without MeSA. Treatments labelled with different letters denote differing levels of statistical significance (F-test; *p* < 0.05) based on the Holm–Sidak method (one-way ANOVA).

The gas chromatography–mass spectrometry (GC–MS) analysis of the headspace collections from the MeSA-treated and control (blank formulation) plants revealed significant qualitative and quantitative changes, with a 3.5-fold increase in the total emitted volatiles of the MeSA (100 mg/L)-treated brassica plants compared to the control (*F*_2,51_ = 4.23192; *p* < 0.19; Figure 4). Additionally, the most volatile compounds were emitted from the brassica plants treated with MeSA (100 mg/L) in significantly higher amounts compared to the MeSA (75 mg/L) and control plants (Table 1).

## 3. Discussion

On reflection, our results showed that the exogenous application of MeSA on brassica crops makes them more resistant to *M. persicae*. The survival and larviposition of the peach potato aphids were reduced on the MeSA-treated plants, and the aphids did not prefer the MeSA-treated plants. Conversely, in the parasitoid fly-off bioassay, *D. rapae* spent a longer time on the MeSA (100 mg/L)-treated plants compared to the control. However, this effect was dependent on the MeSA concentration. For instance, the (75 mg/L) MeSA treatment of brassica plants did not show any significant effects on aphid and parasitoid performance. This is not surprising, because the induction of plant defence can have different activation thresholds that depend on a specific concentration of elicitors [21]. Evidence can be seen for a change in the performance of the aphids on the MeSA-treated and control plants from the headspace sampling. The brassica plants treated with MeSA showed high emissions of volatile organic compounds, which seem to be a potential cause of the reduced aphid performance on the treated plants. It would also be interesting to see in future if applying MeSA with other concentrations can cause similar effects on plants.

In the no-choice cage bioassay, the aphid survival rate was reduced by the MeSA treatment by approximately one-fourth, and approximately half as many aphids died on the treated plants by the 48 and 96 h time intervals, respectively. Similarly, aphid reproduction was significantly reduced by the MeSA treatments at both the 48 and 96 h time intervals. Interestingly, reduced aphid performance was observed with increasing concentrations of MeSA treatments. However, the effectiveness of the MeSA on *M. persicae* performance was reduced after 96 h compared to 48 h. In another experiment, an aphid settlement bioassay revealed that the MeSA-treated plants showed low aphid colonisation compared to control plants. In series 2 and series 3, aphid colonisation was reduced by approximately half on the plants treated with MeSA (100 mg/L); this suggests that the aphids were able to differentiate between the volatile compounds released by the treated and control plants in the BugDorm.

A parasitoid fly-off bioassay was used to investigate the behavioural response of parasitoids on the treated plants after being released. It was assumed that the retention of parasitoids on the plants was more important for effective parasitism than their initial arrival rates [22,23]. Therefore, this experiment was performed. Earlier studies on Brassica [22] have shown that parasitoids spend approximately five times longer on *cis*-Jasmone (CJ)-treated pak choi plants. In the current experiment, parasitoids spent more time on the MeSA-treated plants; however, the difference was not statistically significant. Moreover, since different natural enemy species may show different behavioural responses to MeSA, it remains unclear whether MeSA applications can affect other natural enemies’ ecological function in field studies.

The volatile analysis of headspace collected from the treated and control brassica plants supported the above results by revealing a quantitative difference in volatile emissions. The volatile blend released by MeSA-treated plants particularly (100 mg/L) showed a significant increase in emitted volatile compounds, such as dihydrojasmone, CJ, methyl isothiocyanate, citronellol, α-cedrene, and (E, E)-α-farnesene. Previous studies have shown that most of these emitted volatile compounds have been found to be responsible for altering pest performance on host plants [22,24]. In particular, CJ has been found to be a vital plant-derived volatile compound that induces defence in several crop plants against various sucking pests [22]. Similarly, dihydrojasmone treatments of brassica plants hindered the foraging activity of *M. persicae* [24]. Furthermore, volatile compounds such as (E, E)-α-farnesene, CJ, and methyl salicylate also play a vital role in the recruitment of natural enemies [7,22,25,26].

In previous studies, MeSA has been found to be a potential compound for enhancing plant growth, development, and defence systems, and makes them more tolerant against biotic and abiotic stresses [10,20,27,28]. MeSA plays a critical role in signal transduction by activating defence-related genes that trigger defence responses in plants [29,30]. Several crop plants have been found to show a high level of direct and indirect defence strategies after MeSA treatments, for instance, tobacco [31] rice [30,32], apple [33], soybean [20], barley [34], and tomato [35]. In particular, MeSA treatments stimulate the production of defence enzymes in rice (*Oryza sativa*) [30]. Seed treatments and foliar treatments of MeSA have shown a substantial upregulation of peroxidase (POD) in rice [30]. In poplar trees, different concentrations of MeSA enhance the production of the defence-related enzyme superoxide dismutase (SOD) [36]; furthermore, MeSA treatments of poplar leaves increase the production of the volatile compounds (*Z*)-3-hexen-1-ol and (*Z*)-3-hexenyl acetate [37].

An accumulating body of evidence supports that MeSA treatments of crop plants reduce the performance of rice leaffolder *Cnaphalocrocis medinalis* on rice [16], whitefly *Trialeurodes vaporariorum* on tomato [19], soybean aphid *Aphis glycines* on soybean [20], bird cherry oat aphid *Rhopalosiphum padi* on barley [34], black bean aphid *Aphis fabae* on broad bean [38], western flower thrips *Frankliniella occidenatlis* on aromatic plants (meadow sweet, laurel, and sage) [39,40], and tomato leafminer *Tuta absoluta* on tomato [35]. MeSA alginate beads reduce the abundance of wheat aphid *Sitobion avenae* in wheat [41]. Furthermore, MeSA treatments of crop plants successfully increase the abundance of natural enemies, such as *Scolothrips takahashii* [42]*, Chrysopa nigricornis, Geocorispallens, Stenthorus punctum,* hoverflies [43]*,* and *Oligota kashmirica* [42]. Field plots baited with MeSA cards/lures show a high abundance of *Chrysopa nigricornis* [10]*, Deraeocoris brevis, Stethorus punctum, Hemerobius* sp., and *Orius tristicolor* [7].

Conclusively, our study shows that MeSA treatments of brassica crops induce defence against *M. persicae* aphids by increasing aphid mortality and reducing aphid settlement on treated plants. The current study shows that MeSA has the potential to induce defence in brassica against one of the most threatening sucking pests, *M. persicae.* It remains unclear if MeSA applications affect plant–herbivore–parasitoid interactions in other cultivars or crop species, since plant responses to phytohormone treatments can be species-/cultivar-specific. In further, studies are needed to investigate how MeSA treatments induce significant changes in volatile emissions, for instance, by knocking out the receptors for MeSA. In this study, we found the effects of induced volatile mixtures on insects’ performance, though screening specific functional volatiles from this mixture and clarifying the mechanism through which they affect insects must be studied in future.

## 4. Materials and Methods

### 4.1. Insects

Insect colonies (*M. persicae* and *Diaeretiella rapae*) were originally obtained from Harper Adam University, Shropshire, UK. For experiments, *Myzus persicae* aphids (clone O) were reared on pak choi *Brassica rapa* subsp. *chinensis* (Chinese cabbage) in the insectary in the centre of Applied Entomology and Parasitology at Keele University, UK, under controlled conditions (photoperiod 16L: 8D, 24 °C, 38% RH). Parasitoid *D. rapae* was reared on pak choi plants infested with *M. persicae* under controlled conditions (photoperiod 16 L: 8 D, 20 °C, 40% RH). Upon their emergence, honey solution (1:1 in water) for food was provided to parasitoids. For experiments, only 2–3-days-old, mated females were used.

### 4.2. Plants

Brassica cultivar *Brassica rapa* subsp. *Chinensis* cv. Hanakan (pak choi) was obtained from Warwick University. The effect of MeSA treatment had not been tested previously on this crop. Plants were grown under controlled environmental conditions (20 °C, 33% RH, 16:8 photoperiod) in a growth chamber (MLR-352-PE, Panasonic, The Netherlands). Brassica plants were grown individually in 7.5 cm pots in John Innes No. 2 compost (Westland Horticulture Limited, Tyrone, UK). Plants at BBCH growth stage 14 (five true leaves) were used for the experiments [22,44].

### 4.3. Plant Treatment

Plants were sprayed with an aqueous emulsion of methyl salicylate (MeSA) (Sigma Aldrich, Buchs, Switzerland) as described in [22]. A stock solution was prepared (100 mg/L) from MeSA, and the formulation was dissolved in deionised water [33,45] which was further diluted to make a solution of 75 mg/L. Meanwhile, control plants were treated with deionised water. Effects of both concentrations (100 mg/L and 75 mg/L) were tested by spraying the plants using an Oshide spray bottle (100 mL; Zhengzhou Xinrui Tongda Metal and Material Co., Ltd., Zhengzhou, China) [16,39]. Three trigger pulls of spray formulation (250 μL) were applied to each plant from a distance of 30 cm [22]. Sprayed plants were left for 24 h and then used for experiments. Control plants and MeSA-treated plants were placed in different compartments to avoid any plant–plant interactions.

### 4.4. Aphid Performance Bioassay

Performance of *M. persicae* was assessed on pak choi plants and observations were recorded after 48 h and 96 h. In first series of experiments, aphids were allowed to feed on treated and control plants for 48 h, whereas in second series, aphids were allowed to feed on plants in clip cages for 96 h. For each replicate in each experiment, fresh plants and aphids were used. In each clip cage (2.5 cm in diameter, Bioquip Product Inc., Compton, CA, USA), ten adult alate *M. persicae* were placed, and cages were attached to the lower surface of plant leaves, as described in [22]. Two clip cages were placed on each plant. Fifteen replicates were maintained for each treatment (control, MeSA 75 mg/L and MeSA 100 mg/L). To assess the survival and fecundity of aphids, plants containing clip cages were left undisturbed in a controlled environment room (25 °C, 33 % RH, 16:8 photoperiod). Plants were assessed after 48 h (series 1) and 96 h (series 2). For assessment, leaves containing the clip cages were cut, and cages were removed without losing any aphids. Numbers of live adults and nymphs were recorded.

### 4.5. Aphid Settlement Bioassay

In a choice test bioassay, MeSA-treated and control plants were placed in a Bugdorm insect cage (60 × 60 × 60 cm; NHBS Ltd., Devon, UK) and were kept in a controlled environment room (25 °C, 37 % RH, 16:8 photoperiod). There were three series of experiments; series 1 included control plants and treated plants with MeSA (75 mg/L), series 2 included control plants and treated plants with MeSA (100 mg/L), and series 3 included control and MeSA-treated plants with both concentrations (75 mg/L and 100 mg/L). Each Bugdorm contained four plants (two treated and two control) at alternate positions in Series 1 and 2. While in series 3, three plants were placed in a Bugdorm in a triangular shape at equal distance from the centre. A vial containing 50 alate *M. persicae* was positioned in the centre of the Bugdorm and opened, and then the Bugdorm was left for 24 h. Counts of settled aphids were recorded 24 h after their release. Ten replicates were performed for both series 1 and series 2, while 15 replicates were performed for series 3. The position of treatments was alternated between replicates.

### 4.6. Parasitoid Fly-Off Bioassay

The experiment was adapted from [22], and slightly modified to test MeSA’s effect on parasitoids, in which the foraging time of *D. rapae* females was recorded [21]. Timer was used to record the total spent time by *D. rapae.* In an open-fronted cage, a single female parasitoid was released from a vial onto a plant leaf and then total time spent by *D. rapae* on plant was recorded by direct observation. An observation was terminated when the parasitoid flew away from the plant, which was considered as leaving the foraging ‘patch’. However, a cap of a maximum of 25 min was set for each replicate, and if parasitoid did not leave the plant, replicate was ended after 25 min. Ten replicates of MeSA-treated and control plants were performed for each concentration. Treated and control replicates were observed alternately. All experiments were performed between 9:00 am and 1:30 pm.

### 4.7. Entrainment Collection

Entrainment collection was carried out following a procedure adapted from [22], in which the whole plant was enclosed in an oven bag (35 × 43 cm; Bacofoil, Telford Shropshire, UK, flower seal roasting bag). Each bag was open at the bottom and closed at the top. An outlet was open at the upper end of the bag to Porapak Q filter, whereas bottom part of the bag was closed using a rubber band around the pot. Charcoal-filtered air was pumped out at 600 mL/min and pulled in at 400 mL/min through Porapak filter. Entrainment collection was carried out for a period of 48 h, after which the Porapak filters were eluted with 500 µL of diethyl ether into the sample vials and stored at −20 °C in a freezer for chemical analysis or olfactometer bioassay.

### 4.8. Volatile Analysis

Volatile analysis was performed on a 7820A GC coupled to a 5777B single quad mass selective detector (Agilent Technologies, Cheadle, UK). The gas chromatograph was fitted with a non-polar HP5-MS capillary column (with 30 m × 0.25 mm × 0.25 μm film thickness) coated with (5%-phenyl)-methylpolysiloxane (Agilent Technologies, Santa Clara, CA, USA) and used hydrogen carrier gas at a constant flow rate of 1.2 mL min−1. Automated injections of 1 μL each were made using a G4513A autosampler (Agilent Technologies, Santa Clara, CA, USA) in splitless mode (285 °C), with oven temperature programmed at 35 °C for 5 min then at 10 °C min−1 to 285 °C. Identification of compounds was carried out according to their mass spectra, linear retention index relative to retention time of n-alkanes, and co-chromatography with authentic compounds [22].

### 4.9. Statistical Analyses

Aphid clip cage bioassay—Differences in the mean number of live aphids on control and MeSA-treated plants were compared for Pak choi after 48 h by one-way ANOVA. Prior to analysis, data were examined for a normal distribution using the Shapiro–Wilk test. Comparisons among means were performed using Holm–Sidak method (*p* < 0.05).

Aphid settlement bioassay—Differences in the mean number of aphids settling on control and MeSA-treated plants were compared for pak choi after 48 h using a one-tail paired *t*-test for series 1 and 2, while for series 3 statistical analysis was performed with one-way ANOVA. Prior to analysis, data were examined for a normal distribution using the Shapiro–Wilk test.

Parasitoid foraging bioassay—The total time spent by parasitoid wasps foraging on control and MeSA-treated plants was evaluated by one-way ANOVA. Prior to analysis, data were examined for a normal distribution using the Shapiro–Wilk test. Comparisons among means were performed using Holm–Sidak method (*p* < 0.05).

Volatile analysis—Univariate analysis (F-test) of variances was performed to investigate whether the concentrations of individual volatile compounds differed between treated and untreated brassica plants. All univariate analyses were performed using SigmaPlot 12.3 (Systat Software Inc., San Jose, CA, USA).

## Figures and Tables

**Table 1:** Emissions (in nanogram; mean ± SE; *n* = 5) of volatile released by MeSA-treated and control plants.

Plant Volatiles	*Brassica rapa*			*p*
	Control	75 mg/L	100 mg/L	
Alcohols				
2-ethyl-1-hexanol	0.28 ± 0.21	0.3 ± 0.21	0.9 ± 0.19	0.13
2-butyl-1-octanol	ND	0.1 ± 0.05	0.12 ± 0.04	0.11
Aldehydes				
Decanal	ND	0.02 ± 0.02	0.02 ± 0.01	0.47
Aliphatic hydrocarbons				
Dodecane	0.9 ± 0.66	0.08 ± 0.05	0.1 ± 0.06	0.34
(E)-3-tetradecene	2.34 ± 0.50	1.23 ± 0.50	0.23 ± 0.10	**0.02**
Benzenoids				
Methyl salicylate (MeSA)	0.64 ± 0.57	0.75 ± 0.22	2.58 ± 0.48	**0.03**
Benzothiazole	ND	0.01 ± 0.01	0.4 ± 0.03	0.41
Esters				
cis-3-hexenyl acetate	0.1 ± 0.04	0.3 ± 0.10	0.3 ± 0.19	0.52
2-ethylhexyl acetate	ND	ND	0.1 ± 0.05	0.06
Ketones				
Dihydrojasmone	0.2 ± 0.11	1.2 ± 0.46	3.1 ± 0.79	**0.01**
cis-Jasmone (CJ)	ND	ND	0.5 ± 0.19	**0.02**
N-containing compounds				
Methyl isothiocyanate	ND	ND	0.1 ± 0.04	**0.02**
Benzyl nitrile	0.01 ± 0.01	ND	ND	0.40
Terpenes				
D-limonene	0.1 ± 0.04	0.8 ± 0.40	1.1 ± 0.58	0.31
Citronellol	0.45 ± 0.25	0.8 ± 0.37	4.1 ± 1.06	**0.01**
α-cedrene	ND	0.45 ± 0.20	2.1 ± 0.85	**0.049**
ß-elemene	ND	0.2 ± 0.10	0.2 ± 0.08	0.19
(E, E)-α-farnesene	0.1 ± 0.08	0.1 ± 0.01	2.4 ± 0.99	**0.04**

Plants were treated 24 h before the start of entrainment collection. VOCs are ordered according to increasing retention time under each class. Bold *p*-values denote significant differences (one-way analysis of variance; *p* < 0.05).

## Data Availability

The data that support our findings of this study are available from the corresponding author upon reasonable request.
